# Prevalence of Charcot Foot Among Diabetes Mellitus Patients Under Follow-Up at the Integrated Diabetic Centers of Hospital Kulim and Hospital Raja Permaisuri Bainun: A Cross-Sectional Study

**DOI:** 10.7759/cureus.85708

**Published:** 2025-06-10

**Authors:** Ming Long Kam, Yae Tyug Yap, Hoong Kooi Kok, Kishan Rasiah Seevasoothy, Yan Siew Lim, Ming Yen Hong, Mohamad Izani Ibrahim, Tharumaraja Thiruselvam

**Affiliations:** 1 Orthopaedics, Hospital Raja Permaisuri Bainun, Ipoh, MYS; 2 Pharmacy, Hospital Raja Permaisuri Bainun, Ipoh, MYS; 3 Orthopaedics, Hospital Kulim, Kedah, MYS; 4 Pharmacy, Hospital Kulim, Kedah, MYS

**Keywords:** ankle-brachial systolic index, charcot neuroarthropathy, diabetes mellitus, diabetic neuropathy, multidisciplinary team

## Abstract

Introduction: Charcot foot is a debilitating complication of diabetes mellitus (DM), characterized by joint destruction, deformity, and instability due to neuropathy-induced microtrauma. Despite its severe impact on mobility and quality of life, Charcot foot remains underdiagnosed. This study aims to determine the prevalence of Charcot foot among diabetic patients at two major referral hospitals in Malaysia and identify associated risk factors.

Methods: A cross-sectional study was conducted at the Integrated Diabetic Centers (IDCs) of Hospital Kulim and Hospital Raja Permaisuri Bainun (HRPB). Diabetic patients aged ≥18 years attending follow-ups at these centers were recruited via convenient sampling. Data collection included structured interviews, clinical examinations, laboratory investigations, and radiographic assessments. The diagnosis of Charcot foot was confirmed based on clinical findings, imaging, and laboratory parameters. Statistical analyses, including Fisher's exact test, were conducted using IBM SPSS Statistics for Windows, Version 20.0 (IBM Corp., Armonk, New York, United States), with significance set at p<0.05.

Results: A total of 675 diabetic patients were included, with a mean age of 56.1 years (SD±13.96). Men comprised 58.2% (n=393/675) of participants, while 41.8% (n=282/675) were women. The overall prevalence of Charcot foot among diabetic patients in this study was 1.8% (n=12/675). Gender was significantly associated with Charcot foot (p=0.005), with a higher prevalence among women (3.5%; n=10/282) than men (0.5%; n=2/393). The mean DM duration among Charcot foot patients was 19.67 years (SD±7.34), with 66.7% (n=8/12) having DM for 11-20 years. Poor glycemic control was prevalent, with a mean HbA1c of 9.21% (SD±1.87) and 75% (n=9/12) of Charcot foot patients having HbA1c ≥7%. Additionally, 50% (n=6/12) had a history of diabetic foot ulcers, and 16.7% (n=2/12) had undergone prior amputation.

Conclusion: Charcot foot is a significant but often underdiagnosed complication in diabetic patients, particularly in women and those with long-standing, poorly controlled DM. Early detection and multidisciplinary management are crucial to reducing morbidity. Future research should focus on longitudinal studies to assess disease progression and intervention effectiveness.

## Introduction

Charcot foot is a chronic, debilitating, and destructive condition that affects bones and joints in patients with peripheral neuropathy, most commonly due to diabetes mellitus (DM). It is characterized by varying degrees of joint dislocation, fractures, and deformities, often affecting the foot and ankle. Although the term Charcot neuropathic osteoarthropathy is sometimes used, it more broadly refers to neuropathic joint disease and is not confined to the foot. The majority of patients who develop Charcot foot are in their sixth or seventh decade of life, and over 80% have had DM for more than 10 years [1]. Charcot foot may present with either painless or painful symptoms, depending on the stage of the disease. Pain, when present, is typically seen in the acute inflammatory phase (Eichenholtz stage 0 or 1) and is attributed to underlying bone and joint destruction, microfractures, and active inflammation [2].

The incidence of diabetic Charcot foot has been increasing [3], making early detection and intervention critical. Two primary theories explain the pathophysiology of Charcot foot: the neurovascular theory and the neurotraumatic theory [4]. The neurovascular theory suggests that joint destruction occurs due to increased blood flow to the area, triggered by nerve damage, leading to bone weakening and fractures. In contrast, the neurotraumatic theory proposes that the loss of sensation in the affected area results in repetitive microtrauma, ultimately causing joint destruction. The absence of pain perception due to neuropathy allows mechanical stress and minor injuries to go unnoticed, triggering an inflammatory response that further compromises bone integrity. Because of its non-specific early presentation, Charcot foot is frequently misdiagnosed as cellulitis or osteomyelitis, leading to delays in appropriate treatment and increasing the risk of severe deformities and disability, as noted by Salini et al. and Elsayed et al. [4,5].

DM is a growing global health concern, with its prevalence steadily increasing, particularly in Malaysia. Type 2 diabetes mellitus (T2DM), in particular, is expected to reach pandemic levels by 2030 [5]. As a consequence of multiple pathophysiological changes associated with DM, patients frequently suffer from foot-related complications, including infections, ulcerations, Charcot foot, and gangrene [6]. While diabetic foot ulcers are more commonly recognized, Charcot foot presents an equally serious threat, significantly increasing the risk of lower limb amputation if not diagnosed and managed early, as reported by Elsayed et al. and Huret et al. [5,6]. The diagnosis and management of Charcot foot in DM remain challenging due to its complex nature and the lack of standardized diagnostic protocols.

Studies have shown that the prevalence of Charcot foot among diabetic populations varies widely, largely due to differences in diagnostic criteria, patient awareness, and healthcare accessibility [7]. According to Macfarlane et al., implementing standardized screening protocols and early detection programs is essential for reducing the incidence and severity of Charcot foot in high-risk diabetic patients [8]. Hospital Kulim and Hospital Raja Permaisuri Bainun (HRPB) serve as key referral centers for diabetic foot complications in Malaysia. However, the true prevalence of Charcot foot among their patient populations remains unclear. Identifying the burden of this condition and its associated risk factors is essential for improving screening strategies, optimizing clinical management, and implementing preventive measures.

As suggested by Bullen et al., greater emphasis on early diagnosis, patient education, and multidisciplinary care can significantly reduce the long-term disability associated with Charcot foot [9]. Educating high-risk individuals, particularly those with diabetic peripheral neuropathy, about Charcot foot should be prioritized to improve patient outcomes [10]. This study aims to determine the prevalence of Charcot foot among diabetic patients at these hospitals and to identify the key risk factors contributing to its development. By enhancing our understanding of Charcot foot in the Malaysian healthcare setting, we hope to improve patient outcomes and establish more effective prevention and management strategies.

## Materials and methods

A cross-sectional study was conducted at the Integrated Diabetic Centers (IDCs) of Hospital Kulim and HRPB after obtaining approval from the Medical Research and Ethics Committee (MREC) of the Ministry of Health (MOH) (approval number: 23-00406-AJL). Diabetic patients aged 18 years and above attending follow-ups at these centers were recruited via convenient sampling. Eligibility for inclusion in the study requires a documented diagnosis of T2DM for a duration of no less than five years. This criterion is based on established evidence indicating that Charcot foot most frequently occurs in individuals with long-standing DM. Specifically, the condition is significantly more prevalent among patients with a DM duration exceeding 10 years. Multiple studies have reported that the mean duration of DM at the time of Charcot foot diagnosis typically ranges between 15 and 20 years, underscoring the importance of prolonged hyperglycemic exposure in the pathogenesis of this complication [4]. Accordingly, setting a minimum disease duration of more than five years in this study ensures the study population includes individuals at higher risk for Charcot-related complications, thereby increasing the likelihood of capturing relevant clinical features and enhancing the external validity of the study findings. Furthermore, the exclusion criteria encompassed the absence of DM or radiographic evidence of Charcot foot. Patients with active foot infections, clinically or radiologically confirmed osteomyelitis, or recent antibiotic treatment were excluded to avoid diagnostic ambiguity. Those with severe peripheral arterial disease, evidenced by non-palpable pulses or prior vascular intervention within the past three months, were also excluded. Additional exclusions included patients with non-diabetic causes of neuropathy (e.g., alcoholic, uremic, or hereditary), inflammatory arthropathies such as rheumatoid arthritis, congenital foot deformities, and significant cognitive impairment or psychiatric illness precluding informed consent or protocol adherence.

Data collection included structured interviews, clinical examinations, laboratory investigations, and radiographic assessments. Demographic variables collected included age, gender, ethnicity, body mass index (BMI), education level, pre-exposure education on diabetic foot care, occupation, and adherence to a diabetic diet. Medical history assessment included DM duration, type, treatment regimen (oral hypoglycemic agents, insulin, or combination therapy), DM-related complications (e.g., neuropathy, nephropathy, retinopathy), history of foot ulcers, foot surgery, trauma, and comorbidities (hypertension, dyslipidemia, chronic kidney disease).

Laboratory tests included glycosylated hemoglobin (HbA1c) for glycemic control, erythrocyte sedimentation rate (ESR) for inflammation, white blood cell (WBC) count for infection, and hemoglobin (Hb) and albumin for nutritional and systemic status. Bilateral foot radiographs (anteroposterior and lateral views) were analyzed using the modified Eichenholtz classification and Brodsky anatomical classification.

Comprehensive foot assessments included inspection for swelling, deformity, ulceration, and warmth. Peripheral neuropathy was assessed via a 10 g monofilament test, while pain was evaluated using a standardized pain scale. Vascular status was examined using the ankle-brachial systolic index (ABSI), peripheral pulse examination, and Doppler ultrasound. Charcot foot was diagnosed based on clinical, imaging, and laboratory findings. The progression of Charcot foot was staged using the Eichenholtz classification system, which divides the disease into three primary phases: development, coalescence, and reconstruction [11]. In certain cases, the Sanders and Frykberg classification was also utilized to anatomically localize the affected region within the foot [12]. This system categorizes Charcot foot into five types based on the predominant site of involvement, aiding in more precise anatomical assessment and management planning.

The sample size calculation was performed using the Scalex SP calculator. Based on an expected prevalence of 7% (derived from previous studies reporting a Charcot foot prevalence of between 0.08% and 13% among diabetic populations) [4,13], the required sample size is 626, with a margin error or absolute precision of ±2% in estimating the prevalence of 95% confidence and considering the potential loss/attrition of 5%. With this sample size, the anticipated 95% confidence interval (CI) is between -5% and 9% [14].

Data analysis was performed using IBM SPSS Statistics for Windows, Version 20.0 (IBM Corp., Armonk, New York, United States). Descriptive statistics summarized demographic and clinical characteristics. The prevalence of Charcot foot was reported as a percentage, and associations with risk factors, including age, gender, and race, were analyzed using Fisher's exact test. A p-value of less than 0.05 was considered statistically significant.

## Results

As shown in Table 1, a total of 675 diabetic patients were included in this study, with a mean age of 56.1 years (SD±13.96). A significant proportion of patients were above 60 years old, reflecting a population with long-standing DM and an increased risk of DM-related complications. The gender distribution showed that 58.2% (n=393/675) of participants were male, while 41.8% (n=282/675) were female.

**Table 1:** Demographic characteristics of respondents (n=675) Prevalence of Charcot foot: 1.8% (12 patients) Mean±SD (age): 56.1±13.96

Characteristics	n	%
Age group (years)		
18-30	33	4.9
31-40	60	8.9
41-50	116	17.2
51-60	204	30.2
>60	262	38.8
Gender		
Male	393	58.2
Female	282	41.8
Race		
Malay	400	59.3
Chinese	117	17.3
Indian	158	23.4

The overall prevalence of Charcot foot among diabetic patients in this study was 1.8% (n=12/675). A statistically significant association was observed between gender and Charcot foot occurrence (p=0.005), with a notably higher prevalence among women (3.5%; n=10/282) compared to men (0.5%; n=2/393) (Table 2). This suggests that female diabetic patients may have a greater susceptibility to Charcot foot, possibly due to differences in bone density and hormonal influences. A study published in Diabetes Care found that patients with DM and Charcot foot had a higher risk of osteoporosis compared to those without Charcot foot, with an odds ratio of 1.3 (95% CI 1.1-1.5). Notably, more women than men had osteoporosis in both groups, indicating a gender disparity in bone health among diabetic patients [15]. Research by Ali et al. highlights that estrogen plays a significant role in bone metabolism. Estrogen deficiency, common in postmenopausal women, leads to increased bone resorption and decreased bone mineral density. This hormonal influence may contribute to the higher prevalence of osteoporosis observed in women with DM and Charcot foot [16].

**Table 2:** Demographic characteristics of Charcot foot patients (n=12) DM: diabetes mellitus; OHA: oral hypoglycemic agents; DFU: diabetic foot ulcer Mean±SD (duration of DM): 19.67±7.34 Mean±SD (HbA1C): 9.209±1.8735

Characteristics	n	%
Duration of DM (years)		
≤10	1	8.3
11-20	8	66.7
>20	3	25
Type of diabetic medications		
Insulin	3	25
OHA	2	16.7
Both OHA and insulin	7	58.3
HbA1C (%)		
<7	3	25
≥7	9	75
History of DFU		
Yes	6	50
No	6	50
History of amputation		
Yes	2	16.7
No	10	83.3

Among the 12 patients diagnosed with Charcot foot, the mean duration of DM was 19.67 years (SD±7.34) (Table 2). Most patients (66.7%; n=8/12) had DM for 11-20 years, while 25% (n=3/12) had DM for more than 20 years. This highlights a strong correlation between prolonged DM duration and the development of Charcot foot, reinforcing the need for long-term DM management and foot monitoring in patients with chronic DM.

Poor glycemic control was a prominent feature among Charcot foot patients, with an average HbA1c level of 9.21% (SD±1.87). A significant majority (75%; n=9/12) had HbA1c levels 7% or above, indicating suboptimal DM control. This finding aligns with existing literature linking chronic hyperglycemia to neuropathy and Charcot foot development. Persistent exposure to elevated blood glucose levels may contribute to nerve damage, reduced pain sensation, and progressive foot deformities (Table 2).

Additionally, 50% (n=6/12) of Charcot foot patients had a history of diabetic foot ulcers, suggesting that prior foot complications may predispose patients to Charcot foot (Table 3). Recurrent ulcers or pressure injuries can lead to mechanical stress and repetitive trauma, which, in the presence of peripheral neuropathy, may accelerate joint destruction. Furthermore, 16.7% (n=2/12) of Charcot foot patients had undergone prior ipsilateral ray amputation, emphasizing the severe complications associated with late-stage Charcot foot and the critical need for early identification and preventive strategies.

**Table 3:** Association between Charcot foot and respondents' characteristics n: frequency; ^a^: Fisher's exact test

Characteristics	Charcot foot		P-value ^a ^
	Yes	No	
	n (%)	n (%)	
Age group (years)			
18-30	0	33 (100%)	0.127
31-40	0	60 (100%)	
41-50	5 (4.3%)	111 (95.7%)	
51-60	5 (2.5%)	199 (97.5%)	
>60	2 (0.8%)	260 (99.2%)	
Gender			
Male	2 (0.5%)	391 (99.5%)	0.005
Female	10 (3.5%)	272 (96.5%)	
Race			
Malay	7 (1.8%)	393 (98.2%)	0.165
Chinese	0	117 (100%)	
Indian	5 (3.2%)	153 (96.8%)	

As shown in Table 3, the prevalence of Charcot foot varied across age groups, but no statistically significant association was found between age and Charcot foot occurrence (p=0.127). The highest prevalence was observed among patients aged 41-50 years (4.3%; n=5/116), followed by those aged 51-60 years (2.5%; n=5/204). Interestingly, the prevalence decreased in patients older than 60 years (0.8%; n=2/262), despite this group having the highest number of participants (n=262). This suggests that Charcot foot tends to develop earlier in long-standing DM, potentially influenced by disease duration and metabolic control.

Although Charcot foot was observed across different racial groups, there was no statistically significant association between race and Charcot foot occurrence (p=0.165) (Table 3). The highest prevalence was among Indian patients (3.2%; n=5/158), followed by Malay patients (1.8%; n=7/400), while no cases were reported among Chinese patients (0%; n=0/117). This finding is consistent with the study by Fauzi et al., which reported no significant association between race and the occurrence of Charcot foot among Malay, Indian, and Chinese patients. In their study, 43.8% of patients with Charcot foot were Malay, 50% were Indian, and 6.2% were Chinese [1]. The absence of Charcot foot in the Chinese subgroup may be attributed to genetic factors, lifestyle differences, or healthcare-seeking behaviors, which may contribute to better foot health and DM management. However, given the sample size limitations, further studies are needed to explore racial disparities in Charcot foot development.

These findings suggest that gender is a significant risk factor for Charcot foot, with female patients exhibiting a higher prevalence (Table 3). While age and race were not significantly associated with Charcot foot, trends indicate that diabetic patients aged 41-50 years and individuals of Indian ethnicity may be more affected. These results highlight the importance of early screening, patient education, and targeted preventive strategies, particularly among high-risk groups such as women and diabetic patients aged 41-50 years.

## Discussion

Charcot foot is a well-recognized complication among patients with diabetic neuropathy, with studies demonstrating its increasing prevalence in this population [17]. Similar to the study conducted by Elsayed et al. [5], Fauzi et al. [1] highlighted the significant burden of Charcot foot in diabetic patients, a finding that aligns with previous literature. Various risk factors contribute to the development of diabetic Charcot foot, including obesity, peripheral neuropathy, comorbid renal failure, advanced age (>65 years), and race (White), as identified in a large cohort study [18].

The prevalence of Charcot foot in this study (1.8%) is consistent with international reports, though variations exist due to differences in diagnostic criteria, patient demographics, and healthcare accessibility, as noted by Rogers et al. and Pakarinen et al. [12,17]. These discrepancies highlight the need for standardized diagnostic protocols to ensure the early detection and appropriate management of Charcot foot in diabetic patients. Kumar et al. define DM as a chronic condition characterized by persistently elevated blood glucose levels, significantly impacting patients' quality of life. It is also a major contributing factor to lower extremity complications, including Charcot foot [19]. Furthermore, Charcot foot compromises both the physical health and overall well-being of affected individuals and their families.

Consistent with findings from Rogers et al. [12] and Huret et al. [6], poor glycemic control emerged as a significant factor in the development of Charcot foot, as evidenced by the high mean HbA1c levels observed in this study [17]. Chronic hyperglycemia is a key contributor to neuropathy and vascular dysfunction, both of which heighten the risk of Charcot foot and other DM-related complications. This underscores the need for stringent glycemic control to prevent the onset and progression of Charcot foot. Although specific HbA1c targets for Charcot foot prevention have not been universally defined, general DM management guidelines advocate for an HbA1c target of less than 7% to minimize the risk of complications, including neuropathy and foot ulcers [20].

Early screening, patient education, and glycemic control are fundamental strategies for preventing Charcot foot, as emphasized in studies by Kumar et al. and Svendsen and Jansen [19,21]. Regular foot assessments, particularly for high-risk diabetic patients, facilitate early detection and timely intervention. Additionally, structured education programs focusing on DM self-management, foot hygiene, and appropriate footwear can significantly reduce the risk of foot complications and improve patient outcomes.

Multidisciplinary team (MDT) management is crucial for improving outcomes and reducing the morbidity associated with Charcot foot. MDT involvement has been shown to enhance health outcomes in patients with diabetic foot infections, as highlighted by Choi et al. [2]. A collaborative approach involving endocrinologists, orthopedic surgeons, podiatrists, rehabilitation specialists, and DM educators is essential for optimizing patient care and preventing disease progression. Early detection through routine diabetic foot screening, radiographic assessments, and laboratory investigations enables timely intervention, thereby reducing the risk of severe complications. Additionally, Stancu et al. reported that implementing an MDT approach significantly improves outcomes in diabetic foot ulcer management [22].

Individuals with early-onset DM are often reluctant to seek medical attention, as they perceive themselves to be in good health and may not prioritize dietary or treatment adherence. This behavior frequently leads to poor glycemic control and subsequent DM-related complications [23]. Charcot foot manifests as an acute aseptic inflammation of the bones and joints in the foot. If not diagnosed and managed promptly, it may lead to bone collapse, resulting in deformity, foot ulcers, amputation, and even death, as described by Svendsen and Jansen [21]. Additionally, chronic hyperglycemia contributes to peripheral neuropathy, a key factor in the pathogenesis of Charcot foot [24].

The insidious onset and painless nature of Charcot foot pose significant diagnostic challenges, often leading to delays in diagnosis and treatment, as noted by Amsah et al. [25]. Future research should incorporate longitudinal follow-up studies to better understand the progression of Charcot foot and evaluate the long-term effectiveness of various treatment approaches, as suggested by Bullen et al. and Bansod et al. [26,27]. Further research comparing conservative management with surgical interventions could provide valuable insights into optimizing treatment strategies and improving patient outcomes.

This study has several limitations. First, as a cross-sectional study, it captures data at a single time point, limiting the ability to assess disease progression or establish causation between risk factors and Charcot foot development. Second, the study relied on convenient sampling, which may introduce selection bias and limit the generalizability of the findings to the broader diabetic population. Additionally, the diagnosis of Charcot foot was based on clinical and radiographic assessments, which, while widely used, may lack the sensitivity of more advanced imaging techniques such as MRI [28].

Furthermore, data on certain risk factors, such as levels of physical activity and specific dietary habits, were not included, which could have provided additional insights into Charcot foot risk. Future studies should aim to address these limitations by employing prospective study designs, larger sample sizes, and more comprehensive diagnostic tools to enhance understanding of Charcot foot and improve patient outcomes.

## Conclusions

This study reinforces the importance of early screening and timely intervention strategies in mitigating the long-term impact of Charcot foot. Given the strong correlation between poor glycemic control, prolonged DM duration, and the development of Charcot foot, patient education on DM management, lifestyle modifications, and foot care practices should be emphasized. Structured foot care programs, including offloading strategies and customized footwear, play a crucial role in preventing ulceration and further deformity. Additionally, routine monitoring of at-risk individuals can facilitate the early detection of Charcot foot, reducing complications and improving overall patient outcomes.

Future research should focus on the long-term outcomes of Charcot foot management, particularly in the Malaysian population, where genetic predisposition, lifestyle factors, and healthcare accessibility may influence disease progression. Comparative studies evaluating different treatment modalities, including conservative management versus surgical intervention, are essential in determining the most effective approach for patient care. Furthermore, advancements in diagnostic tools, such as novel imaging techniques and biomarkers, could enhance the early identification of Charcot foot in high-risk patients, thereby improving clinical decision-making and treatment outcomes.

In conclusion, an integrated multidisciplinary approach, combined with early screening, patient education, and effective DM management, remains essential in reducing the burden of Charcot foot. Strengthening healthcare provider awareness and implementing evidence-based guidelines will be crucial in improving patient outcomes and preventing long-term disability associated with this debilitating condition.
